# Production of Basal Bodies in bulk for dense multicilia formation

**DOI:** 10.12688/f1000research.8469.1

**Published:** 2016-06-28

**Authors:** Xiumin Yan, Huijie Zhao, Xueliang Zhu

**Affiliations:** 1 State Key Laboratory of Cell Biology, CAS Centre for Excellence in Molecular Cell Science, Institute of Biochemistry and Cell Biology, Shanghai Institutes for Biological Sciences, Chinese Academy of Sciences, Shanghai, China

**Keywords:** ciliogenesis, centriole assembly, Deuterosome, deuterosome-dependent

## Abstract

Centriole number is normally under tight control and is directly linked to ciliogenesis. In cells that use centrosomes as mitotic spindle poles, one pre-existing mother centriole is allowed to duplicate only one daughter centriole per cell cycle. In multiciliated cells, however, many centrioles are generated to serve as basal bodies of the cilia. Although deuterosomes were observed more than 40 years ago using electron microscopy and are believed to produce most of the basal bodies in a mother centriole-independent manner, the underlying molecular mechanisms have remained unknown until recently. From these findings arise more questions and a call for clarifications that will require multidisciplinary efforts.

## Introduction

**Figure 1.**
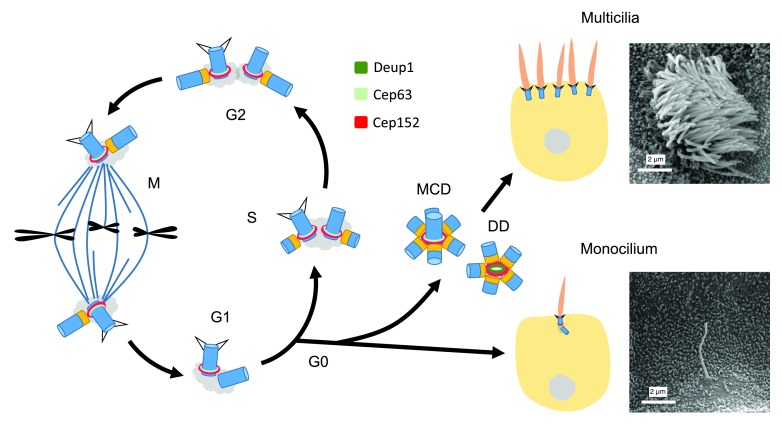
Centriole biogenesis and cilia formation. The centrosome in a G1 cell contains a pair of mother-daughter centrioles. Upon entering the S phase, each centriole starts to duplicate one daughter centriole so that the centriole number remains constant after mitosis ( **a**). When the cell enters G0, the mother centriole can be transformed into the basal body to support monocilium formation ( **b**). Alternatively, both the mother centriole-dependent (MCD) and deuterosome-dependent (DD) pathways can be activated to generate an abundance of centrioles for dense multicilia formation ( **c**). The scanning electron microscopy images show a primary cilium in the collecting duct of mouse kidney and multicilia of a multiciliated cell in mouse tracheal epithelium, respectively. Centrioles are drawn in blue and their cartwheels in orange.

The centriole is a cylinder-shaped organelle that serves as the core of the centrosome or the basal body of the cilium ^1– 5^. Nascent centriole formation usually depends on pre-existing mother centrioles. Normally in one cell cycle each mother centriole produces only one daughter centriole, that is directly adjacent ( Figure 1). Such tight control ensures proper mitosis, since only two centrosomes are required to function as the spindle poles. It also guarantees that the centriole number remains constant after cell division ( Figure 1).

Ciliogenesis occurs at the G0 or G1 stage of the cell cycle ( Figure 1) ^1, 3, 6^. In vertebrates, most cells can possess a primary cilium, which functions as a sensory organ for diverse environmental signals. Mammalian epithelial tissues such as those lining the inner surface of the trachea, the oviduct, and the brain ventricles, however, have abundant multiciliated cells (MCCs) with hundreds of cilia ( Figure 1). These multicilia are motile and their beating is critical for mucus clearance, ovum transport, or cerebrospinal fluid circulation ^7^. How then do such cells generate sufficient numbers of basal bodies?

The mystery was initially uncovered by electron microscopy (EM) on a variety of MCC-containing tissues in the 1960’s and 1970’s. The mother centriole was observed to be surrounded by multiple daughter centrioles in MCCs. Moreover, many granular or ring-shaped EM structures termed deuterosomes (this name will be used in this review), procentriole precursor bodies, dense granules, and generative complexes were also able to initiate procentriole assembly ^8– 12^. Importantly, the deuterosomes were estimated to produce most of the basal bodies required. Nevertheless, it is only recently that we have begun to understand the molecular mechanisms involved, which will be the major focus of this review.

## Mother centriole-dependent centriole assembly

Tremendous progress has been made toward understanding how a daughter centriole is born in cycling cells. A group of proteins, including Cep152 and Cep63, are specifically located around the proximal side of the mother centriole. In the G1 phase, the polo-like kinase PLK4 binds to Cep152 to form the site of centriole assembly ^13– 17^. In the S phase, a cartwheel structure is formed at the PLK4 site, followed by the assembly of the nine sets of microtubule triplets and other components of the daughter centriole. Centriole assembly is completed by the G2 phase and, following mitosis, each daughter cell inherits a mother-daughter pair of centrioles ( Figure 1) ^1– 5^.

Interestingly, mother centrioles in cycling cells are capable of generating more than one daughter centriole. For instance, overexpression of PLK4 results in multiple PLK4 foci around the mother centriole and overproduction of daughter centrioles ^18, 19^. Overexpression of Cep152 or the cartwheel proteins SAS-6 or STIL also has a similar effect ^20– 23^. These observations not only indicate that cycling cells execute the one-daughter-centriole-per-mother rule by restricting the levels of several critical proteins but also suggest that MCCs may break this rule by simply upregulating the protein levels. Indeed, when mouse tracheal epithelial cells (MTECs) are induced to form multicilia, they express high levels of these proteins ^19, 24, 25^. The importance of PLK4 and Cep152 in mother centriole-dependent (MCD) centriole overduplication of MTECs is also verified ^19^.

## Deuterosome-dependent centriole assembly

**Figure 2.**
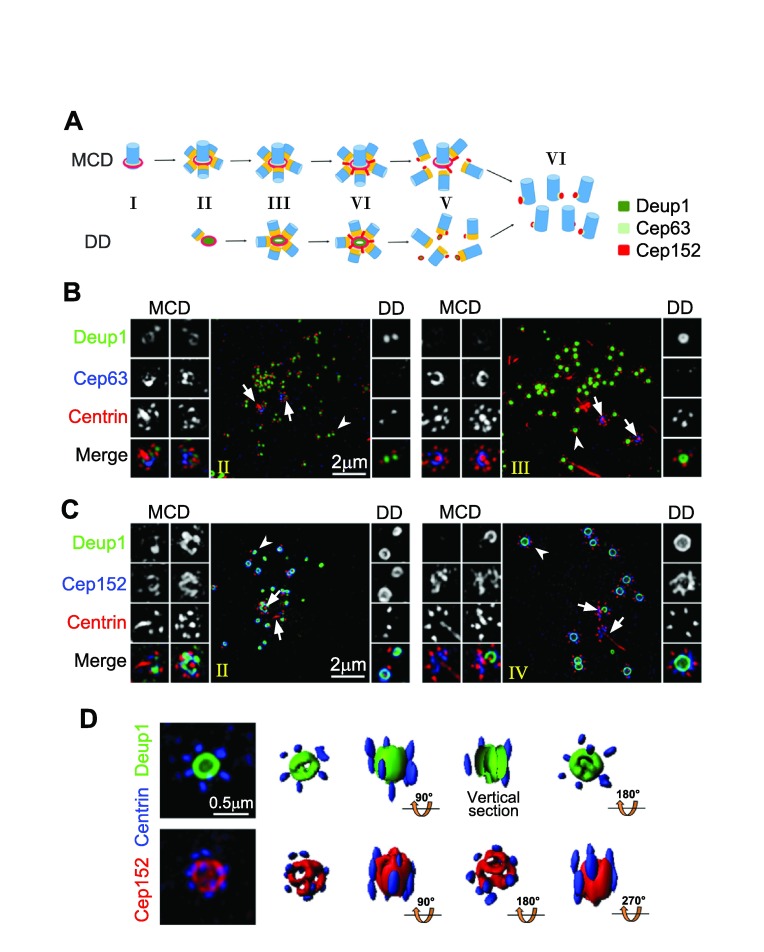
Centriole amplifications in mouse tracheal epithelial cells (MTECs) and mouse ependymal cells (MEPCs). ( **A**) Illustration for centriole amplification stages in MTECs ^19^. Centrioles are drawn in blue and their cartwheels in orange. ( **B**) Three-dimensional structured illumination microscopy (3D-SIM) images for MTECs at early stages (II and III) of centriole amplification. MTECs cultured as described previously ^19^ were immunostained for Deup1, Cep63, and Centrin and imaged using a DeltaVision OMX V3 microscopic system (GE Healthcare). The mother centrioles (arrows) and representative deuterosomes (arrowheads) are magnified 2× to show details. ( **C**) 3D-SIM images showing centriole amplification in MEPCs. MEPCs were isolated from neonatal C57BL/6J mice and cultured as described ^32^. The cells were fixed at day three after serum starvation and immunostained for Deup1, Cep152, and Centrin. The stages (II and IV) are defined as in the MTECs. Note that MEPC deuterosomes ( **C**) are usually much larger than those in MTECs ( **B**). ( **D**) SIM images of two large MEPC deuterosomes immunostained for Deup1 and Centrin (top row) or Cep152 and Centrin (bottom row). Their 3D profiles are also shown. Abbreviations: DD, deuterosome dependent; MCD, mother centriole dependent.

The discovery of an essential deuterosome component, Deup1 (also called Ccdc67), has promoted the understanding of deuterosome-dependent (DD) centriole biogenesis. Strikingly, Deup1 is a paralog of Cep63 ^19^. Cep152 binds to both Cep63 and Deup1 to stabilize them and be recruited, respectively, to the mother centriole and the deuterosome ^19, 26^. Therefore, if we consider the Cep63-Cep152-containing proximal ring of the mother centriole as a platform, or ‘cradle’, that supports nascent centriole assembly, deuterosomes are analogous cradles, independent of mother centrioles ( Figure 2A). In MTECs, deuterosomes appear initially as foci with zero to two associated procentrioles ( Figure 2A-B, stage II). Their sizes then enlarge, accompanied by an increase in procentriole numbers ( Figure 2A-B, stage III). They are disassembled upon completion of centriole assembly ( Figure 2A) ^19, 27^. Usually 50–100 deuterosomes can be found in a MTEC, sufficient for the production of hundreds of centrioles ( Figure 2B) ^19^. Mouse ependymal cells (MEPCs) displayed a similar centriole amplification process, but their deuterosomes are usually much larger in size and smaller in number ( Figure 2C).

The beauty of such a DD pathway is obvious: cycling cells only need to turn off the DD pathway by shutting down *Deup1* expression to avoid the production of extra centrioles. On the other hand, as MCCs are terminally differentiated and no longer able to enter the cell cycle, turning on the DD pathway and upregulating other genes critical for basal body assembly can safely fulfill their demand on large numbers of basal bodies. For instance, the Multicilin-E2F4/5 complex is known to activate the transcription of *Deup1*, *Plk4*, *Cep152*, and many other centriolar protein genes in MCCs ^28– 30^. Other proteins such as cyclin O appear to fine-tune the transcription program ^31^.

## Deuterosome structures and components

Deuterosome size varies remarkably in different tissues and species: for instance, from 100–200 nm (diameter) in rat or mouse MTECs ^8, 19^ to more than 500 nm in the mouse oviduct ^10^. Larger deuterosomes are capable of supporting more procentrioles. As deuterosomes look mostly ring shaped in transmission EM, they were proposed to be roughly sphere shaped, capable of assembling centrioles in all directions ^8, 10^. Serial ultra-thin sections of MEPCs support this notion ^32^.

Three-dimensional profiling of subdiffraction images from both MTECs and MEPCs, however, suggests that Deup1 and Cep152 are arranged in a ring-shaped configuration in the deuterosome, with the Cep152 signals enwrapping those of Deup1 from outside ( Figure 2C) ^19^. Such a configuration is topologically analogous to the mother centriole cradle. Only the ends of the deuterosome appear relatively amorphous. For instance, in large deuterosomes such as those of MEPCs, the Cep152 signals may exhibit several ‘holes’ at each end ( Figure 2D). Procentrioles tend to be assembled on the outer wall of the deuterosome but can be found at both ends as well ( Figure 2D) ^19^.

Whether there are additional proteins to construct the outer wall, fill the center, or cap the ends of the deuterosome is presently unknown. Ccdc78, a coiled coil domain-containing protein, is reported as a deuterosome-specific protein required for centriole amplification in the *Xenopus* embryonic epidermis ^33^. Nonetheless, mouse Ccdc78, expressed either endogenously or exogenously, was not detected on Deup1-positive deuterosomes in our hands, raising the possibility that Ccdc78 may be either an amphibian-specific deuterosome component or even not a *bona fide* one.

## Deuterosome assembly

How deuterosome components are packed together to form the supramolecular structure is also an important issue. Fibrous granules (also called fibrogranular material or proliferative elements), clouds of material abundant in 40 to 80 nm granules that coincide with deuterosome formation in MCCs, were proposed to be precursors of the deuterosome ^8– 10^. PCM-1, a component of fibrous granules, however, failed to show deuterosome localization ^34^. Its depletion by RNA interference also didn’t impair centriole amplification ^25^. Likewise, neither Deup1 nor Cep152 exhibited obvious fibrous granule-like distributions ( Figure 2B-C) ^19^. Since small deuterosomes tend to emerge in bulk and then grow in synchrony and ectopic expression of Deup1 in cycling cells is sufficient to induce the formation of functional deuterosomes ( Figure 2B) ^19^, we propose that deuterosomes can be assembled spontaneously ( Figure 2A).

Interestingly, a recent publication argues for a totally different mechanism ^32^. Based mainly on studies in MEPCs, a model is proposed in which an unknown mechanism recruits Deup1, Ccdc78, and other cradle proteins to a site in the cradle of the young mother centriole to initiate the assembly of both the deuterosome and the daughter centrioles. The deuterosome-procentriole halo is then released so that the site can begin the next assembly cycle. After the release of the last halo, procentrioles on all the deuterosomes start to elongate and mature. Thus, both the deuterosome formation and the massive centriole biogenesis are MCD processes. Deuterosomes function merely as shuttles to carry the daughter centrioles away from their mother centriole into the cytoplasm ^32, 35^.

This model, despite its uniqueness, still needs further verification. Firstly, it remains to be shown whether this is the sole and universal way of deuterosome generation. Deup1 is capable of mother centriole localization ( Figure 2B-C) ^19^. It is thus understandable that some of the protein there may serve as seeds to initiate deuterosome assembly. Since live imaging in the MEPCs suggests that the generation of one halo requires about two hours ^32^, such an efficiency would demand several days to generate the 50–100 deuterosomes in MTECs, while the entire centriole amplification process takes roughly only one day ^19, 32^. Thus, both the spontaneous and MCD pathways may contribute. Furthermore, there might be multiple deuterosome nucleation sites on both the young and the old mother centrioles ( Figure 2B-C). Secondly, the model is apparently incompatible with the observation that the numbers of deuterosome-associated procentrioles increase over time ( Figure 2B-C) ^19^. Even if one or two daughter centrioles could be carried away from the mother centriole by each nascent deuterosome, their subsequent increase in numbers still argues for the existence of *de novo* DD centriole biogenesis. Finally, what defines the deuterosome nucleation site on the mother centriole and how the cytoplasmic halos can wait until the last one is released are also issues for future clarification.

## Conservation of the deuterosome-dependent pathway

Phylogenetic analysis suggests that *Deup1* is originated from a common fish ancestor of the lobe-finned fish and tetrapods in the vertebrate evolution to boost cilia density in MCCs ^19, 36^. Accordingly, in contrast to the lobe-finned fish (such as lungfish), MCCs of the ray-finned fish (such as zebrafish), which have no *Deup1*, ** contain only sparse cilia ^37, 38^. Many invertebrates, however, possess MCCs with dense multicilia ^39– 42^. Deuterosome-like ultrastructures have also been reported in some invertebrate species ^43, 44^. A comprehensive knowledge of strategies for centriole amplification throughout metazoan evolution will thus require an understanding of the mechanisms for multiciliogenesis in the invertebrate.

## Conclusions and perspectives

The mechanism of centriole amplification is both exciting and challenging. Because the sizes of centrioles and deuterosomes are below or close to the optical diffraction limit, technical limitations of imaging are a current major bottleneck restraining studies of centriole amplification in MCCs. Although 3D structured illumination microscopy (SIM) ^45^ has proven its power in the past ^19, 32^, the development and introduction of super-resolution techniques with higher spatial (especially the z-axis) and temporal resolutions ^46– 49^ are expected to greatly facilitate studies in the field. Furthermore, other cutting-edge techniques such as cryo-electron tomography, omics analysis, and computational biology may help to solve issues on the structure, formation, growth, disassembly, and function of the deuterosome as well as the entire mechanism that controls appropriate on-and-off switching of the centriole amplification program.

## Abbreviations

DD, deuterosome dependent; EM, electron microscopy; MCC, multiciliated cell; MCD, mother centriole dependent; MEPC, mouse ependymal cell; MTEC, mouse tracheal epithelial cell; SIM, structured illumination microscopy.

## References

[1] Nigg EA Stearns T: The centrosome cycle: Centriole biogenesis, duplication and inherent asymmetries. *Nat Cell Biol.* 2011;13(10):1154–60. 10.1038/ncb2345 21968988 PMC3947860

[2] Bornens M: The centrosome in cells and organisms. *Science.* 2012;335(6067):422–6. 10.1126/science.1209037 22282802

[3] Ishikawa H Marshall WF: Ciliogenesis: building the cell's antenna. *Nat Rev Mol Cell Biol.* 2011;12(4):222–34. 10.1038/nrm3085 21427764

[4] Avidor-Reiss T Gopalakrishnan J: Building a centriole. *Curr Opin Cell Biol.* 2013;25(1):72–7. 10.1016/j.ceb.2012.10.016 23199753 PMC3578074

[5] Gönczy P: Towards a molecular architecture of centriole assembly. *Nat Rev Mol Cell Biol.* 2012;13(7):425–35. 10.1038/nrm3373 22691849

[6] Goetz SC Anderson KV: The primary cilium: a signalling centre during vertebrate development. *Nat Rev Genet.* 2010;11(5):331–44. 10.1038/nrg2774 20395968 PMC3121168

[7] Brooks ER Wallingford JB: Multiciliated cells. *Curr Biol.* 2014;24(19):R973–82. 10.1016/j.cub.2014.08.047 25291643 PMC4441396

[8] Sorokin SP: Reconstructions of centriole formation and ciliogenesis in mammalian lungs. *J Cell Sci.* 1968;3(2):207–30. 5661997 10.1242/jcs.3.2.207

[9] Anderson RG Brenner RM: The formation of basal bodies (centrioles) in the Rhesus monkey oviduct. *J Cell Biol.* 1971;50(1):10–34. 10.1083/jcb.50.1.10 4998200 PMC2108422

[10] Dirksen ER: Centriole morphogenesis in developing ciliated epithelium of the mouse oviduct. *J Cell Biol.* 1971;51(1):286–302. 10.1083/jcb.51.1.286 5111878 PMC2108250

[11] Kalnins VI Porter KR: Centriole replication during ciliogenesis in the chick tracheal epithelium. *Z Zellforsch Mikrosk Anat.* 1969;100(1):1–30. 10.1007/BF00343818 5354183

[12] Steinman RM: An electron microscopic study of ciliogenesis in developing epidermis and trachea in the embryo of *Xenopus laevis.* *Am J Anat.* 1968;122(1):19–55. 10.1002/aja.1001220103 5654501

[13] Cizmecioglu O Arnold M Bahtz R: Cep152 acts as a scaffold for recruitment of Plk4 and CPAP to the centrosome. *J Cell Biol.* 2010;191(4):731–9. 10.1083/jcb.201007107 21059844 PMC2983070

[14] Hatch EM Kulukian A Holland AJ: Cep152 interacts with Plk4 and is required for centriole duplication. *J Cell Biol.* 2010;191(4):721–9. 10.1083/jcb.201006049 21059850 PMC2983069

[15] Sonnen KF Schermelleh L Leonhardt H: 3D-structured illumination microscopy provides novel insight into architecture of human centrosomes. *Biol Open.* 2012;1(10):965–76. 10.1242/bio.20122337 23213374 PMC3507176

[16] Bettencourt-Dias M Rodrigues-Martins A Carpenter L: SAK/PLK4 is required for centriole duplication and flagella development. *Curr Biol.* 2005;15(24):2199–207. 10.1016/j.cub.2005.11.042 16326102

[17] Habedanck R Stierhof YD Wilkinson CJ: The Polo kinase Plk4 functions in centriole duplication. *Nat Cell Biol.* 2005;7(11):1140–6. 10.1038/ncb1320 16244668

[18] Kleylein-Sohn J Westendorf J Le Clech M: Plk4-induced centriole biogenesis in human cells. *Dev Cell.* 2007;13(2):190–202. 10.1016/j.devcel.2007.07.002 17681131

[19] Zhao H Zhu L Zhu Y: The Cep63 paralogue Deup1 enables massive *de novo* centriole biogenesis for vertebrate multiciliogenesis. *Nat Cell Biol.* 2013;15(12):1434–44. 10.1038/ncb2880 24240477

[20] Dzhindzhev NS Yu QD Weiskopf K: Asterless is a scaffold for the onset of centriole assembly. *Nature.* 2010;467(7316):714–8. 10.1038/nature09445 20852615

[21] Strnad P Leidel S Vinogradova T: Regulated HsSAS-6 levels ensure formation of a single procentriole per centriole during the centrosome duplication cycle. *Dev Cell.* 2007;13(2):203–13. 10.1016/j.devcel.2007.07.004 17681132 PMC2628752

[22] Vulprecht J David A Tibelius A: STIL is required for centriole duplication in human cells. *J Cell Sci.* 2012;125(Pt 5):1353–62. 10.1242/jcs.104109 22349705

[23] Arquint C Sonnen KF Stierhof YD: Cell-cycle-regulated expression of STIL controls centriole number in human cells. *J Cell Sci.* 2012;125(Pt 5):1342–52. 10.1242/jcs.099887 22349698

[24] Hoh RA Stowe TR Turk E: Transcriptional program of ciliated epithelial cells reveals new cilium and centrosome components and links to human disease. *PLoS One.* 2012;7(12):e52166. 10.1371/journal.pone.0052166 23300604 PMC3534086

[25] Vladar EK Stearns T: Molecular characterization of centriole assembly in ciliated epithelial cells. *J Cell Biol.* 2007;178(1):31–42. 10.1083/jcb.200703064 17606865 PMC2064416

[26] Sir JH Barr AR Nicholas AK: A primary microcephaly protein complex forms a ring around parental centrioles. *Nat Genet.* 2011;43(11):1147–53. 10.1038/ng.971 21983783 PMC3299569

[27] Tang TK: Centriole biogenesis in multiciliated cells. *Nat Cell Biol.* 2013;15(12):1400–2. 10.1038/ncb2892 24296417

[28] Ma L Quigley I Omran H: Multicilin drives centriole biogenesis via E2f proteins. *Genes Dev.* 2014;28(13):1461–71. 10.1101/gad.243832.114 24934224 PMC4083089

[29] Balestra FR Gönczy P: Multiciliogenesis: multicilin directs transcriptional activation of centriole formation. *Curr Biol.* 2014;24(16):R746–9. 10.1016/j.cub.2014.07.006 25137586

[30] Stubbs JL Vladar EK Axelrod JD: Multicilin promotes centriole assembly and ciliogenesis during multiciliate cell differentiation. *Nat Cell Biol.* 2012;14(2):140–7. 10.1038/ncb2406 22231168 PMC3329891

[31] Funk MC Bera AN Menchen T: *Cyclin O (Ccno)* functions during deuterosome-mediated centriole amplification of multiciliated cells. *EMBO J.* 2015;34(8):1078–89. 10.15252/embj.201490805 25712475 PMC4406653

[32] Al Jord A Lemaître AI Delgehyr N: Centriole amplification by mother and daughter centrioles differs in multiciliated cells. *Nature.* 2014;516(7529):104–7. 10.1038/nature13770 25307055

[33] Klos Dehring DA Vladar EK Werner ME: Deuterosome-mediated centriole biogenesis. *Dev Cell.* 2013;27(1):103–12. 10.1016/j.devcel.2013.08.021 24075808 PMC3816757

[34] Kubo A Sasaki H Yuba-Kubo A: Centriolar satellites: molecular characterization, ATP-dependent movement toward centrioles and possible involvement in ciliogenesis. *J Cell Biol.* 1999;147(5):969–80. 10.1083/jcb.147.5.969 10579718 PMC2169353

[35] Meunier A Spassky N: Centriole continuity: out with the new, in with the old. *Curr Opin Cell Biol.* 2016;38:60–7. 10.1016/j.ceb.2016.02.007 26924800

[36] Amemiya CT Alföldi J Lee AP: The African coelacanth genome provides insights into tetrapod evolution. *Nature.* 2013;496(7445):311–6. 10.1038/nature12027 23598338 PMC3633110

[37] Kramer-Zucker AG Olale F Haycraft CJ: Cilia-driven fluid flow in the zebrafish pronephros, brain and Kupffer's vesicle is required for normal organogenesis. *Development.* 2005;132(8):1907–21. 10.1242/dev.01772 15790966

[38] Kemp A: Role of epidermal cilia in development of the Australian lungfish, *Neoceratodus forsteri* (Osteichthyes: Dipnoi). *J Morphol.* 1996;228(2):203–21. 10.1002/(SICI)1097-4687(199605)228:2<203::AID-JMOR9>3.0.CO;2-5 29852685

[39] Basti L Endo M Segawa S: Prevalence and intensity of pathologies induced by the toxic dinoflagellate, *Heterocapsa circularisquama*, in the Mediterranean mussel, *Mytilus galloprovincialis*. *Aquat Toxicol.* 2015;163:37–50. 10.1016/j.aquatox.2015.03.012 25840278

[40] Azimzadeh J Wong ML Downhour DM: Centrosome loss in the evolution of planarians. *Science.* 2012;335(6067):461–3. 10.1126/science.1214457 22223737 PMC3347778

[41] Good MJ Stommel EW Stephens RE: Mechanical sensitivity and cell coupling in the ciliated epithelial cells of *Mytilus edulis* gill. An ultrastructural and developmental analysis. *Cell Tissue Res.* 1990;259(1):51–60. 10.1007/BF00571429 2297786

[42] Aono K Fusada A Fusada Y: Upside-down gliding of Lymnaea. *Biol Bull.* 2008;215(3):272–9. 10.2307/25470711 19098148

[43] Ferraguti M Fascio U Boi S: Mass production of basal bodies in paraspermiogenesis of Tubificinae (Annelida, Oligochaeta). *Biol Cell.* 2002;94(2):109–15. 10.1016/S0248-4900(02)01185-1 12148240

[44] Ash BM Stephens RE: Ciliogenesis during the sequential formation of molluscan gill filaments. *Dev Biol.* 1975;43(2):340–7. 10.1016/0012-1606(75)90033-0 1126564

[45] Schermelleh L Carlton PM Haase S: Subdiffraction multicolor imaging of the nuclear periphery with 3D structured illumination microscopy. *Science.* 2008;320(5881):1332–6. 10.1126/science.1156947 18535242 PMC2916659

[46] Schermelleh L Heintzmann R Leonhardt H: A guide to super-resolution fluorescence microscopy. *J Cell Biol.* 2010;190(2):165–75. 10.1083/jcb.201002018 20643879 PMC2918923

[47] Vicidomini G Moneron G Han KY: Sharper low-power STED nanoscopy by time gating. *Nat Methods.* 2011;8(7):571–3. 10.1038/nmeth.1624 21642963

[48] Li D Shao L Chen BC: ADVANCED IMAGING. Extended-resolution structured illumination imaging of endocytic and cytoskeletal dynamics. *Science.* 2015;349(6251):aab3500. 10.1126/science.aab3500 26315442 PMC4659358

[49] Xu K Zhong G Zhuang X: Actin, spectrin, and associated proteins form a periodic cytoskeletal structure in axons. *Science.* 2013;339(6118):452–6. 10.1126/science.1232251 23239625 PMC3815867

